# A randomized interventional clinical trial assessing the safety and effectiveness of PeaNoc XL tablets in managing joint pain and inflammation in arthritis patients

**DOI:** 10.12688/f1000research.138477.1

**Published:** 2023-07-27

**Authors:** Nandakumar Kadanangode Narayanaswam, Eric Caston, Rajappan Chandra Satish Kumar, Thangavel Mahalingam Vijayakumar, Vishagan Sulur Vanangamudi, Negi Pankaj, Abdul Sukkur

**Affiliations:** 1 Apex Laboratories Pvt Ltd, Chennai and Department of Medical Research, SRM Medical College Hospital and Research Centre, SRM Institute of Science and Technology, Kattankulathur, Tamil Nadu, India; 2 Fuji Chemicals, Tokyo, Japan; 3 Clinical Trial & Research Unit, Interdisciplinary Institute of Indian System of Medicine, SRM Institute of Science and Technology, Kattankulathur, Tamil Nadu, India; 4 Department of Pharmacy Practice, College of Pharmacy, SRM Institute of Science & Technology, Kattankulathur, Tamil Nadu, India; 5 Apex Laboratories Private Limited, Chennai, India

**Keywords:** arthritis, pain, palmitoylethanolamide, astaxanthin, inflammation, joint pain

## Abstract

**Background**: Globally, alternative medicine is used widely by most patients for several health challenges. To evaluate the effectiveness and safety of PeaNoc XL Tablet in managing pain and inflammation, a randomized clinical trial and systematic study was designed. PeaNoc XL Tablet has been widely utilized for pain and inflammation management, but no previous studies have examined its efficacy and safety.

The aim of this study was to determine the clinical effectiveness and safety profile of PeaNoc XL in patients with arthritis experiencing joint pain and inflammation.

**Methods**: A randomized, controlled, and an open-label trial was conducted. A total of 155 patients (18 to 60 years) with arthritis were enrolled for participation. Using computer-generated random sequences, the study population was divided into two groups in a randomized manner. Group A received Standard therapy and Group B received Standard therapy with PeaNoc XL Tablet 400mg (two tablets OD after food).

**Results**: Out of 155 patients, a total of 83 individuals were excluded from the study, leaving 72 patients who were randomly assigned to either Group A (n=36) or Group B (n=36). The administration of PeaNoc XL as an adjunct to standard therapy resulted in a significant reduction in levels of TNF-α (P<0.01), IL-1β (P<0.001), IL-6 (P<0.01), and CRP (P<0.01) in arthritis patients experiencing joint pain and inflammation. Conversely, no notable differences were observed from the baseline in the standard therapy group.

**Conclusions**: After 12 weeks of supplementation of PeaNoc XL tablets, as an add-on therapy helps in the reduction of pain score, joint stiffness, and physical stiffness.

**Trial registration: ** CTRI/2022/10/046693.

## Introduction

Alternative medicine, encompassing herbal medicines, phytonutrients, ayurvedic products, and nutraceuticals, is commonly employed by a substantial number of patients worldwide to address diverse health conditions. According to available data, approximately 80% of the global population relies on herbal products as their primary choice of treatment for various ailments. The utilization of alternative medicines, including classical herbal products, has exhibited a consistent and notable upward trend over the past few decades. ^1^

PeaNoc XL Tablet is extensively utilized for the purpose of pain and inflammation management. PeaNoc XL Tablet is a combination of palmitoylethanolamide 400mg and astaxanthin 1mg.

Palmitoylethanolamide (PEA) is an endogenous fatty acid amide belonging to the class of lipid mediators and the family of N-acylethanolamines (NAEs). It shares similarities with the endocannabinoid anandamide (AEA). PEA demonstrates inhibitory effects on the release of pro-inflammatory mediators from activated mast cells and diminishes mast cell activation at nerve injury sites. ^2^ These mechanisms are associated with the alleviation of allodynia and hyperalgesia in the neuropathic pain model.

Astaxanthin is a carotenoid primarily present in natural sources and marine organisms, including microalgae, salmon, trout, krill, shrimp, crayfish, and crustaceans. ^3^ Carotenoids, including astaxanthin, are recognized for their antioxidant properties and have gained significant attention for their therapeutic advantages in conditions related to aging and various diseases.

To the best of our knowledge, there is no scientific study investigating the efficacy and safety of PeaNoc XL in arthritis patients. Therefore, a clinical trial was conducted to assess the clinical efficacy and safety of PeaNoc XL specifically in arthritis patients experiencing joint pain and inflammation.

## Objectives

1. To evaluate the efficacy of PeaNoc XL tablets on biochemical parameters in arthritis patients with joint pain and inflammation.
2. To evaluate the efficacy of PeaNoc XL tablets on inflammatory cytokines in arthritis patients with joint pain and inflammation.
3. To assess the efficacy and safety of PeaNoc XL tablets in relation to the arthritis Index (WOMAC) in arthritis patients with joint pain and inflammation.
4. To evaluate adverse drug reactions in the study period.

## Methods

### Study design

A randomized, controlled, open-labelled trial was conducted at a SRM Medical College Hospital and Research Centre in Tamil Nadu.

### Ethical considerations

The study adhered to the guidelines set forth by the International Committee on Harmonization on Good Clinical Practice and followed the revised version of the Declaration of Helsinki. Approval for the study protocol was obtained from the institutional human ethics committee of SRM Medical College Hospital and Research Centre on March 25, 2022, with Ethics Clearance No: 8275/IEC/2022. The study is registered with the Clinical Trials Registry India (CTRI) under the reference number CTRI/2022/10/046693.

### Investigational product

PeaNoc XL Tablet contains astaxanthin (1mg) + palmitoylethanolamide (400mg)

Direction: two tablets once daily (OD)

### Inclusion criteria

• Eligible participants diagnosed with arthritis, regardless of sex, in the age range of 18 to 60 years (inclusive).
• Patients with a DAS 28 (Disease Activity Score) score greater than or equal to 3.2
• Voluntary willingness to provide written informed consent for participation.
• Ability to comprehend the nature and objectives of the study and demonstrate a willingness to adhere to study procedures.

### Exclusion criteria

• Participants taking other non-steroidal anti-inflammatory drugs (NSAIDs)/painkillers other than standard drug
• Abnormal results on liver function test
• Patients with diabetic neuropathy
• Patients with severe renal, hepatic, cardiac, gastrointestinal, neurological, hematological, or respiratory disorder
• Patients with a psychiatric disorder
• BMI >35 kg/m ^2^ or <20 kg/m ^2^
• Participants who were likely to have surgery during the study period
• Participants who have partaken in any clinical study or clinical trial in the previous 12 weeks
• Known hypersensitivity to the study drugs
• Patients with severe infection
• History of intake of any ayurvedic/herbal/homeopathic/dietary supplements in the last two months
• Pregnant or nursing mothers and women of childbearing age refusing to use contraceptives

### Treatment groups

In this pilot study conducted over a duration of nine months, a sample size of 30 patients was divided into two groups. Group A received standard therapy for the specified condition, while Group B received standard therapy in addition to PeaNoc XL Tablet 400mg, taken orally after food.

Group A: Standard therapy

Group B: Standard Therapy + PeaNoc XL Tablet 400mg (2 Tablets OD after food)

Study Duration - nine months

Sample size - Pilot sample (30 patients in each group) as per WHO guidelines

### Study procedure

The included study populations based on study criteria were randomized into two groups by using a computer-generated random sequence in R software. Group A received Standard therapy and Group B received Standard therapy with PeaNoc XL Tablet 400mg (two tablets OD after food).

The hematology, liver, and kidney functions were evaluated for both groups. The levels of inflammatory cytokines, including TNF-α, IL-1β, IL-6, and CRP, were measured using the enzyme-linked immunosorbent assay (ELISA) technique. Western Ontario and McMaster Universities Arthritis Index (WOMAC) ^4^ questionnaire was assessed subjectively before and after the treatment to evaluate the efficacy of the PeaNoc XL supplementation.

### Biochemical assessment

Morning blood samples of 5mL were collected via venous puncture from participants who had fasted overnight, specifically between the hours of 8:00 a.m. and 10:00 a.m. Following collection, centrifugation was performed using an Eppendorf Centrifuge 5430R. The resulting samples were divided into aliquots and stored at -20°C until analysis. All biochemical tests were conducted using a fully automated clinical chemistry analyzer (EM 360; Transasia, ERBA Diagnostics [Transasia]) with ERBA diagnostics kits (ERBA Diagnostics Mannheim GmbH).

The levels of TNF-α, IL-6, IL-1β, and CRP were quantified utilizing a two-step sandwich-type immunoassay known as the Human Leptin Quantikine ELISA Kit, which incorporates enzymatic amplification.

### WOMAC questionnaire

The WOMAC pain scale is extensively used in the evaluation of hip and knee osteoarthritis (OA). It is a self-managed questionnaire consisting of 24 items divided into three subscales, as follows:

1. Pain (five items): while walking, using stairs, in bed or rest, sitting or lying, and standing upright
2. Stiffness (two items): after the first walk and later in the day
  Physical Function (17 items): climbing stairs, walking, rising from a sitting position, standing, bending down, moving in or out of a vehicle, shopping, putting on or taking off socks, rising from bed, lying in bed, getting in or out of the bath, getting on or off the toilet, doing heavy domestic duties, or light domestic duties. ^5^

The results of the questions are scored on a 0-4 scale:

▪ None (0),
▪ Mild (1),
▪ Moderate (2),
▪ Severe (3), and
▪ Extreme (4)

The scores for each subscale are added up, with a possible score range of

▪ 0-20 for Pain,
▪ 0-8 for Stiffness, and
▪ 0-68 for Physical Function.

Higher scores on the WOMAC signify worse pain, stiffness, and functional limitations.

## Results

**Figure 1.**
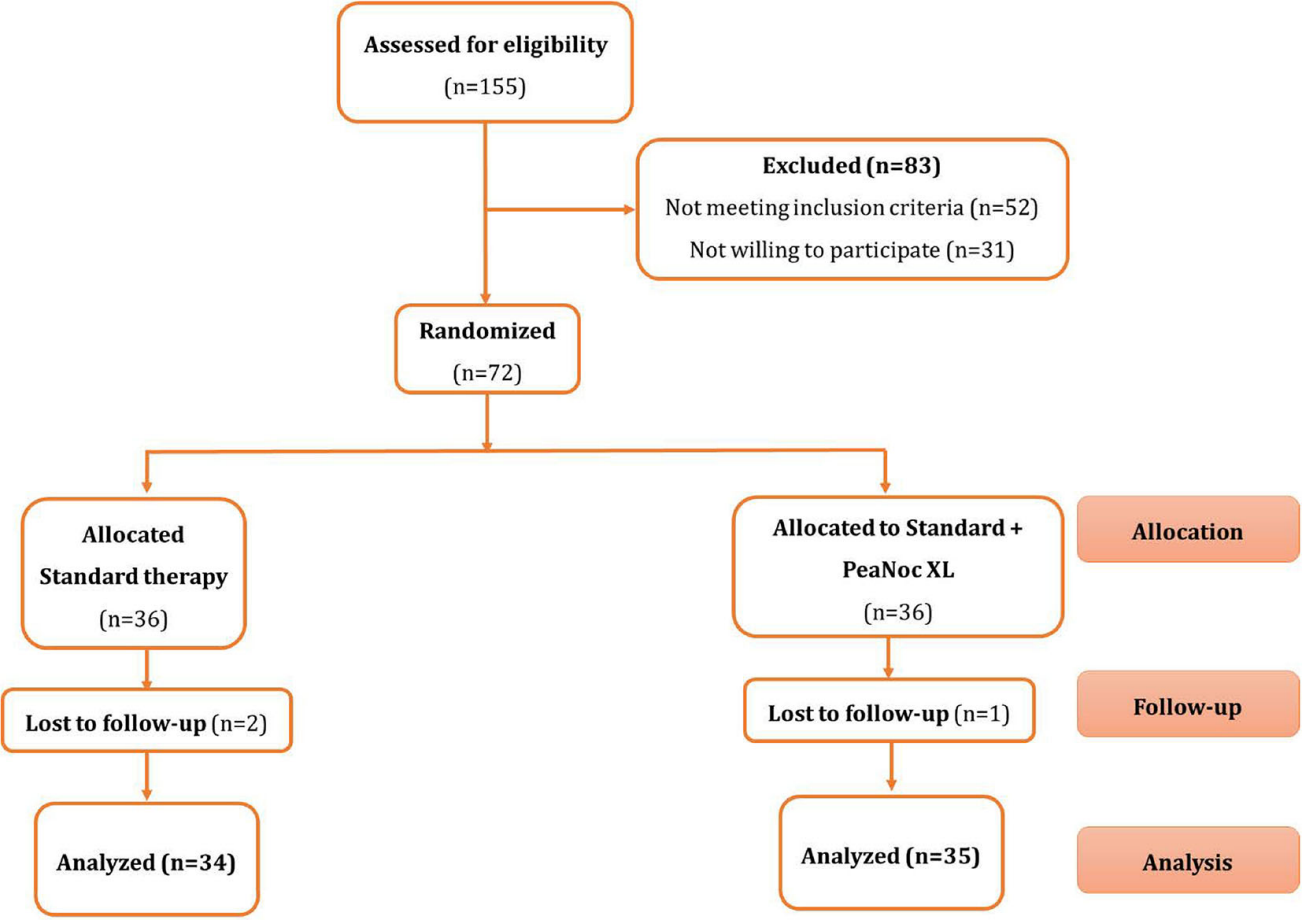
Flow diagram following CONSORT guidelines.

A total number of 155 patients were assessed for admissibility, while 83 patients were excluded. In the end, 72 patients were included and were randomized into two groups. Group A received Standard therapy and Group B received standard therapy with PeaNoc XL ( Figure 1). The baseline characteristics for both groups are mentioned in Table 1.

**Table 1.** Comparison of baseline characteristics between group A and Group B patients.

Parameters	Study groups		P-value
	Group A	Group B	
Age	45.83 ± 6.68	41.73 ± 9.85	0.64
BMI	28.12 ± 4.61	29.63 ± 6.27	0.29
Hip circumference	37.47 ± 2.33	35.40 ± 1.33	0.25
Waist circumference	36.8 ± 1.51	36 ± 0.58	0.08
Platelets	3.11 ± 0.57	3.54 ± 0.53	0.40
Systolic BP	126.67 ± 11.55	126.67 ± 11.55	1.26
Diastolic BP	81.33 ± 8.60	81.33 ± 8.60	1.87
Pulse	86.73 ± 5.96	88.07 ± 5.21	0.36
SPO2	98.23 ± 1.25	99.03 ± 1.25	0.16
Total. bilirubin	0.75 ± 1.39	0.75 ± 1.39	0.96
Direct. bilirubin	0.43 ± 0.13	0.43 ± 0.13	0.77
Indirect. bilirubin	0.62 ± 0.35	0.62 ± 0.35	0.27
Total protein	6.81 ± 0.99	6.81 ± 0.99	0.69
Albumin	4.11 ± 0.59	4.06 ± 0.66	0.74
SGOT	22.87 ± 7.0	22.87 ± 7.0	0.51
SGPT	27.87 ± 6.08	30.27 ± 4.56	0.80
Globulin	2.51 ± 0.46	2.51 ± 0.46	0.67
GGT	27.83 ± 5.26	23.07 ± 5.19	0.17
ALP	124.50 ± 24.32	128.27 ± 31.13	0.60
Serum creatinine	0.8 ± 0.23	0.8 ± 0.23	0.43

(Mean±SD); P>0.05 indicates non-significance.

**Figure 2.**
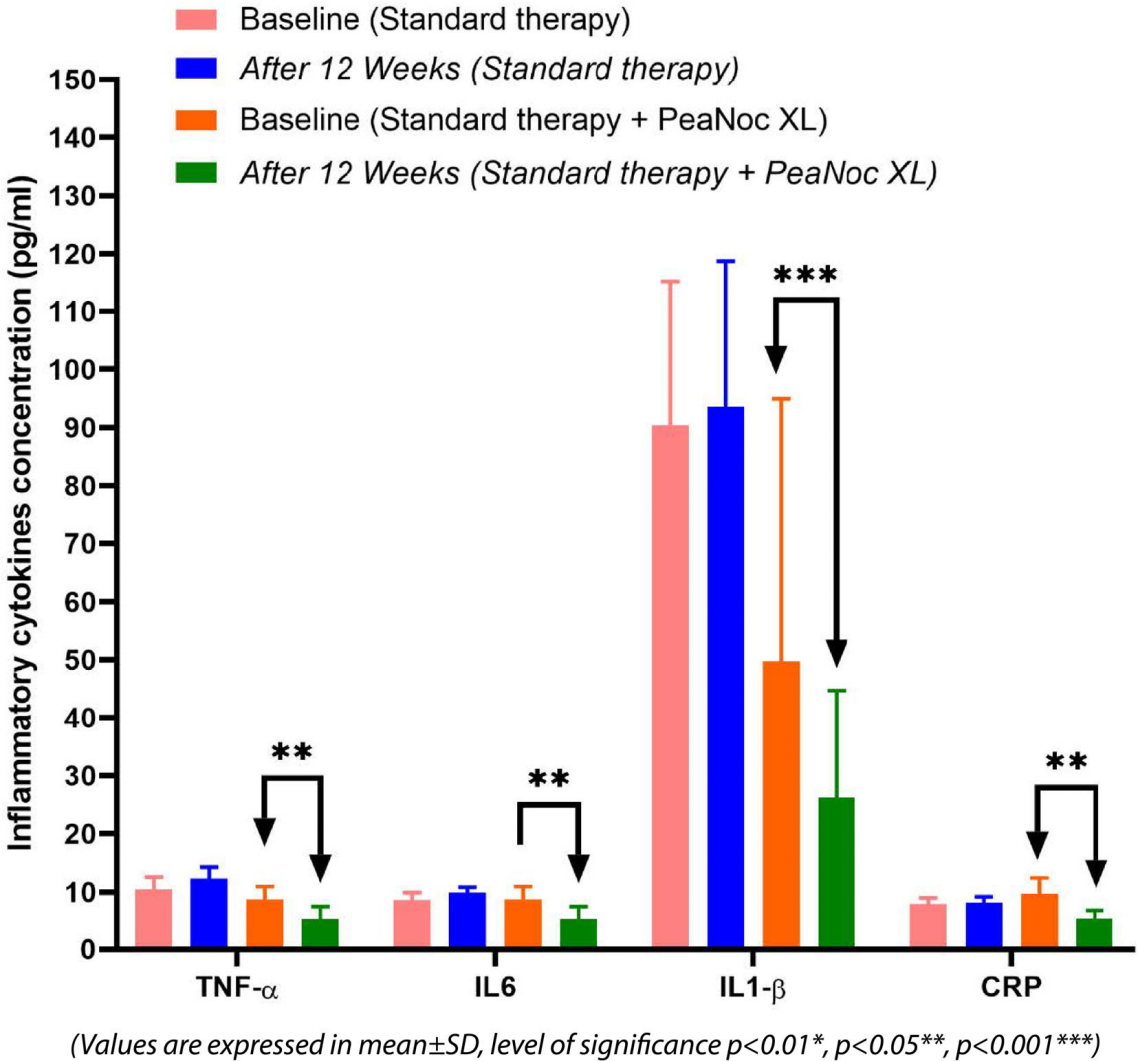
Effect of standard therapy and PeaNoc XL add-on to standard therapy.

**Figure 3.**
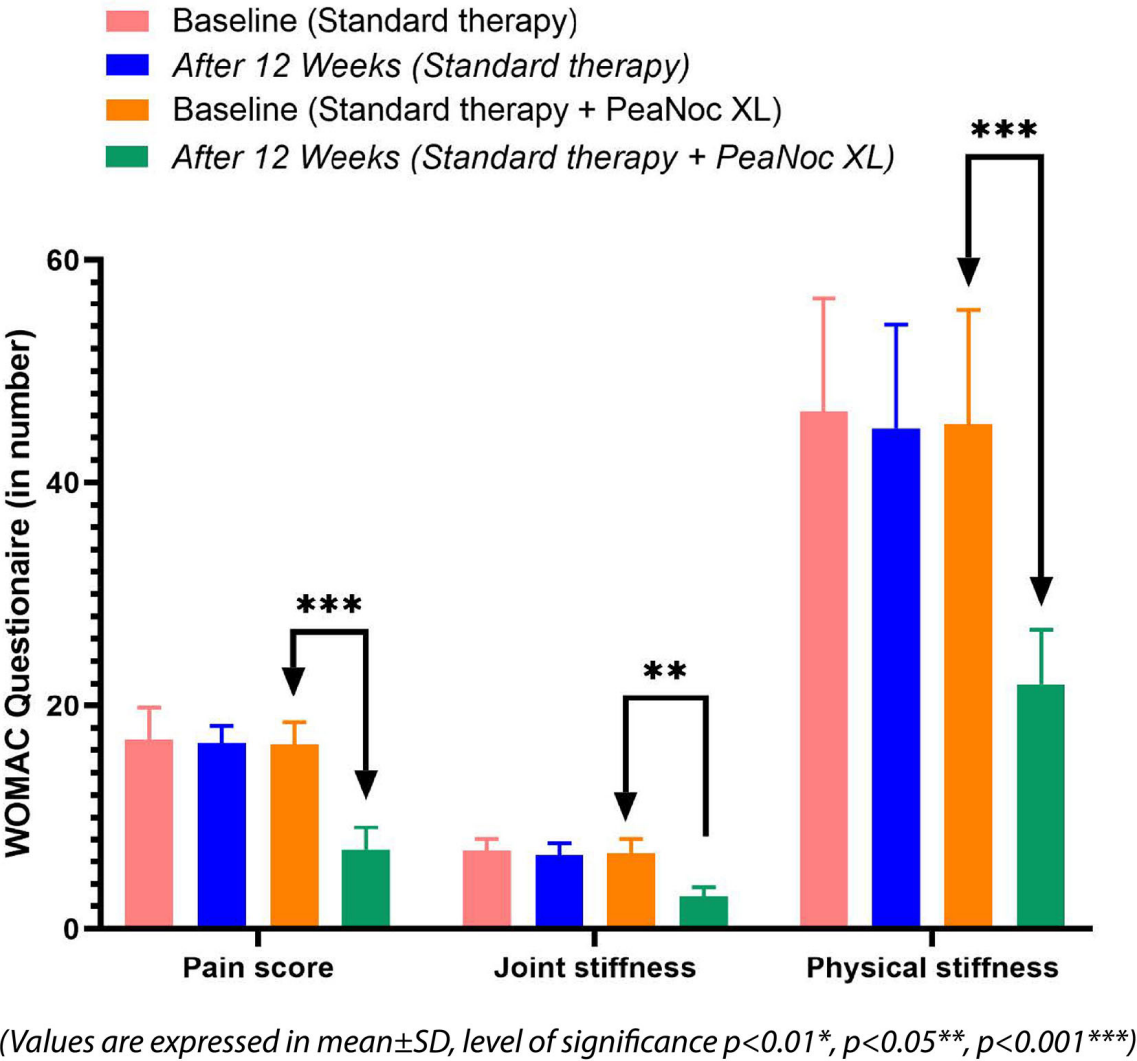
Effect of Standard and PeaNoc XL add-on therapy on WOMAC questionnaire. The values are presented as mean±SD, and the level of significance is indicated as follows: P<0.01* (significant), P<0.05** (significant), and P<0.001*** (highly significant).

In the Standard therapy group (Group A), two patients were lost to follow-up due to non-response to phone calls. In the Standard therapy group + PeaNoc XL group (Group B), one patient was lost to follow-up due to migration. Thus, 34 patients’ data were analyzed in Group A and 35 patients’ data were analyzed in Group B ( Figures 2 and 3). ^6^

Patient demographics were recorded for both groups after receiving informed consent. In the baseline assessment, no significant differences (P>0.05) were observed between age, BMI, hip circumference, waist circumference, platelets, blood, and biochemical parameters among the groups ( Table 1).

Blood samples were obtained from the patients at the beginning of the study (baseline) and at the end of the three-month period for both study groups. These samples were collected to conduct liver and kidney function tests, which included the analysis of various parameters such as total bilirubin, direct bilirubin, total protein, albumin, globulin, serum glutamic-oxaloacetic transaminase (SGOT), serum glutamic pyruvic transaminase (SGPT), gamma-glutamyl transferase (GGT), alkaline phosphatase (ALP), and serum creatinine.

Results showed that PeaNoc XL tablets did not influence any of the parameters. Thus, the safety of the PeaNoc XL is established with the biochemical parameters as shown in Table 2.

**Table 2.** Effect of standard and PeaNoc XL on biochemical parameters.

Parameters	Group A (Standard)		Group B (PeaNoc XL)	
	Baseline	Following 12 weeks of treatment	Baseline	Following 12 weeks of treatment
Total bilirubin	0.75 ± 1.39	0.79 ± 0.27	0.75 ± 1.39	0.79 ± 0.27
Direct bilirubin	0.43 ± 0.13	0.43 ± 0.12	0.43 ± 0.13	0.43 ± 0.12
Indirect bilirubin	0.62 ± 0.35	0.65 ± 0.20	0.62 ± 0.35	0.65 ± 0.20
Total protein	6.81 ± 0.99	7.16 ± 0.75	6.81 ± 0.99	7.16 ± 0.75
Albumin	4.11 ± 0.59	4.08 ± 0.65	4.06 ± 0.66	4.04 ± 0.67
SGOT	22.87 ± 7.0	23.97 ± 6.27	22.87 ± 7.0	22.97 ± 6.27
SGPT	27.87 ± 6.08	27.83 ± 5.32	30.27 ± 4.56	23.63 ± 7.46*
Globulin	2.51 ± 0.46	2.90 ± 0.49	2.51 ± 0.46	2.90 ± 0.49
GGT	27.83 ± 5.26	29.63 ± 3.85	23.07 ± 5.19	15.67 ± 5.63
ALP	124.50 ± 24.32	124.07 ± 18.74	128.27 ± 16.13	102.3 ± 58.8
Serum creatinine (mg/dL)	0.8 ± 0.23	1.12 ± 0.24	0.7 ± 0.14	1.04 ± 0.13

Values are presented as mean±SD. Statistical significance levels are indicated as follows: P<0.01* (significant), P<0.05** (significant), P<0.001*** (highly significant), and P>0.05 indicating non-significance.

### Efficacy of PeaNoc XL tablets on inflammatory cytokines

The values are presented as mean±SD. Significance levels are denoted as follows: P<0.01* (significant), P<0.05** (significant), and P<0.001*** (highly significant).

The levels of inflammatory cytokines, including TNF-α, IL-6, IL-1β, and CRP, were assessed using the ELISA technique. The addition of PeaNoc XL to standard therapy resulted in a significant reduction in the levels of TNF-α (P<0.01), IL-6 (P<0.01), IL-1β (P<0.001), and CRP (P<0.01) among patients experiencing joint pain and inflammation. In contrast, the standard therapy group did not exhibit any notable difference compared to the baseline levels of these cytokines.

### Safety and efficacy of PeaNoc XL tablets on arthritis index (WOMAC)

WOMAC questionnaire has three main areas such as pain score, joint stiffness, and physical stiffness. The addition of PeaNoc XL as an adjunct therapy demonstrated significant reductions in pain (P<0.001), joint stiffness (P<0.01), and physical stiffness (P<0.001) compared to baseline among patients with joint pain and inflammation. In contrast, standard therapy did not exhibit statistically significant differences in these parameters in the same patient population.

***Safety assessment***

Following a 12-week treatment with PeaNoc XL therapy, all hematological and biochemical safety parameters remained within normal ranges. No severe adverse side effects were reported during the study period, and none of the patients withdrew their consent due to adverse drug reactions.

## Discussion

PEA is a bioactive lipid mediator resembling endocannabinoids that falls within the NAE fatty acid amide family. It is ubiquitously present in various tissues, including the brain. PEA is thought to be synthesized in response to cellular injury as a pro-homeostatic protective mechanism and its production is enhanced in disease conditions. PEA exhibits diverse effects, encompassing anti-inflammatory, analgesic, antimicrobial, antipyretic, antiepileptic, immunomodulatory, and neuroprotective properties. ^7^

The multifaceted mechanisms of action exhibited by PEA offer potential therapeutic advantages in various disorders, including allergic reactions, the common cold, chronic pain, joint pain, and neurodegenerative conditions. Additionally, PEA has been shown to enhance muscle recovery and improve cognition, mood, and sleep. ^7^

PEA presents itself as a promising alternative for the relief of joint pain. A clinical trial employing a triple-blind, randomized, parallel-arm design revealed that PEA exhibited superior efficacy in reducing temporomandibular joint (TMJ) OA pain when compared to ibuprofen. ^8^

In a double-blind, randomized, placebo-controlled trial, the efficacy of PEA in patients with mild to moderate knee OA was established. Over the course of an eight-week clinical study, patients who received a high-bioavailability form of PEA demonstrated dose-dependent improvements in joint pain, stiffness, and function, as evaluated using the WOMAC questionnaire. Notably, joint pain decreased by 40% with a 300 mg PEA dosage and by 49.5% with a 600 mg PEA dosage, accompanied by a significant decrease in the use of rescue medication by the end of the eighth week. These findings highlight the potential of PEA as a novel treatment for alleviating pain and related symptoms associated with knee OA. ^9^

The available studies, along with the existing literature, highlight the intriguing potential of PEA as an alternative treatment for addressing joint pain.

Astaxanthin, a red carotenoid pigment present in diverse plant and marine organisms like shrimps and salmon, exhibits a range of biological functions. Notably, it is recognized for its potent antioxidant, anti-inflammatory, and anti-cancer properties. ^10^

A study has provided evidence supporting the potential beneficial effects of astaxanthin in the context of OA. Notably, astaxanthin demonstrated significant reductions in the activities of MMP-1, MMP-3, and MMP-9, as well as inhibited the expression of MMP-1, MMP-2, MMP-9, and MMP-13 in various cell types. These findings are particularly valuable in terms of inhibiting matrix degradation. Consequently, the study suggests that astaxanthin holds promise as an agent with anti-osteoarthritic properties. ^10^

## Key findings

The findings of the present randomized, open-label interventional trial showed that PeaNoc XL tablets help in the reduction of pain, joint stiffness, and physical stiffness (WOMAC questionnaire).

Inflammatory levels of TNF-α, IL1-β, IL-6, and CRP levels in patients with joint pain and inflammation were significantly reduced in PeaNoc XL add-on treatment as compared to baseline and standard therapy.

A 12- week supplementation of PeaNoc XL add-on therapy was effective in arthritis patients with joint pain and inflammation.

PeaNoc XL was well-tolerated as an add-on oral therapy, suggesting its safety and efficacy.

## Conclusions

This randomized study showed that adding PeaNoc XL Tablet to standard therapy effectively reduced pain, joint stiffness, and physical stiffness in arthritis patients. It also resulted in significant reductions in inflammatory markers. PeaNoc XL Tablet demonstrates potential as a safe and effective option for managing joint pain and inflammation in arthritis patients.

## Author contributions

All authors, including K. N. Nandakumar, Dr. E. Caston, Dr. R. C. Satish Kumar, Dr. T.M. Vijayakumar, S. V. Vishagan, Pankaj Negi, and A. Sukkur, made equal contributions to this report. They had full access to all the data in the study and took responsibility for ensuring the integrity and accuracy of the data analysis.

Study design: K. N. Nandakumar, Dr. E. Caston, Dr. R. C. Satish Kumar, Dr. T.M. Vijayakumar, S. V. Vishagan, Pankaj Negi, and A. Sukkur.

Study supervision: K. N. Nandakumar, Dr. E. Caston, Dr. R. C. Satish Kumar, Dr. T.M. Vijayakumar, S. V. Vishagan, Pankaj Negi, and A. Sukkur.

Acquisition, analysis, or interpretation of data **:** K. N. Nandakumar, Dr. E. Caston, Dr. R. C. Satish Kumar, Dr. T.M. Vijayakumar, S. V. Vishagan, Pankaj Negi, and A. Sukkur.

## Data Availability

Figshare: master sheet peanoc xl 2.xlsx, https://doi.org/10.6084/m9.figshare.23641023. ^11^ Figshare: CONSORT checklist for “A randomized interventional clinical trial assessing the safety and effectiveness of PeaNoc XL tablets in managing joint pain and inflammation in arthritis patients”, https://doi.org/10.6084/m9.figshare.23641023. ^11^ Data are available under the terms of the Creative Commons Attribution 4.0 International license (CC-BY 4.0).
