# Supplementary material for: A randomized interventional clinical trial assessing the safety and effectiveness of PeaNoc XL tablets in managing joint pain and inflammation in arthritis patients
Source: F1000Res. 2023 Jul 27;12:895. [Version 1] doi: 10.12688/f1000research.138477.1 (PMC10521040; doi:10.12688/f1000research.138477.1)
Supplement: Peer review history

# Reviewer report: Reviewer response for version 1

**DOI:** 10.5256/f1000research.151678.r194023

**Published:** 2023-09-15

**Authors:** Arnab Karmakar

**Affiliations:** 1 Department of Orthopaedics, Institute of Post Graduate Medical Education and Research, Seth Sukhlal Karni Memorial Hospital, Kolkata, West Bengal, India

This paper is done very accurately and very useful for clinical practice. Pain relief is very important, this paper reflects the proper usage of peanoc-xl and the potency of the medication. The generated data will be useful for future doctors to use the medicine in treating different chronic painful conditions.

Is the work clearly and accurately presented and does it cite the current literature?

Yes

If applicable, is the statistical analysis and its interpretation appropriate?

Yes

Are all the source data underlying the results available to ensure full reproducibility?

Yes

Is the study design appropriate and is the work technically sound?

Yes

Are the conclusions drawn adequately supported by the results?

Yes

Are sufficient details of methods and analysis provided to allow replication by others?

Yes

Reviewer Expertise:

Orthopaedics, pain management

I confirm that I have read this submission and believe that I have an appropriate level of expertise to confirm that it is of an acceptable scientific standard.

# Reviewer report: Reviewer response for version 1

**DOI:** 10.5256/f1000research.151678.r194026

**Published:** 2023-09-06

**Authors:** Shovan Kumar Rath

**Affiliations:** 1 Department of Anesthesiology, Hitech Medical College, Bhubaneswar, Odisha, India

Adding PeaNoc XL Tablet to standard therapy effectively reduced pain, joint stiffness, and physical stiffness in arthritis patients. It must be supplemented for all backpain & arthritis patients. This study adequately shows the effectiveness of PeaNoc XL tablets as an adjunct for chronic pain management in arthritis patients. PeaNoc XL tablets, as an add-on therapy helps in the reduction of pain score, joint stiffness, and physical stiffness.

Is the work clearly and accurately presented and does it cite the current literature?

Yes

If applicable, is the statistical analysis and its interpretation appropriate?

Yes

Are all the source data underlying the results available to ensure full reproducibility?

Yes

Is the study design appropriate and is the work technically sound?

Yes

Are the conclusions drawn adequately supported by the results?

Yes

Are sufficient details of methods and analysis provided to allow replication by others?

Yes

Reviewer Expertise:

pain management

I confirm that I have read this submission and believe that I have an appropriate level of expertise to confirm that it is of an acceptable scientific standard.
